# Composite phaeochromocytomas—a systematic review of published literature

**DOI:** 10.1007/s00423-021-02129-5

**Published:** 2021-03-02

**Authors:** K. Dhanasekar, V. Visakan, F. Tahir, S. P. Balasubramanian

**Affiliations:** 1 University of Sheffield, Sheffield, UK; 2 Newcastle University, Newcastle upon Tyne, UK; 3 Sheffield Teaching Hospitals NHS Trust UK, Sheffield, UK

**Keywords:** Phaeochromocytoma, Ganglioneuroma, Ganglioneuroblastoma, Neuroblastoma, Schwannoma, Adrenal, Incidentaloma, Composite tumours

## Abstract

**Introduction:**

Composite phaeochromocytoma is a tumour containing a separate tumour of neuronal origin in addition to a chromaffin cell tumour. This study reports on two cases from a single centre’s records and presents a systematic literature review of composite phaeochromocytomas.

**Methods:**

In addition to describing 2 case reports, a systematic search of the Medline database from inception up to April 2020 was done for human case reports on composite phaeochromocytomas. Relevant titles and/or abstracts were screened, and full texts were reviewed to identify appropriate studies. Data was extracted and a descriptive analysis of presentation, clinical features, management strategies and outcomes was performed. The quality of included studies was assessed using a critical appraisal checklist.

**Results:**

There were 62 studies included, with a total of 94 patients. Of 91 patients where data was available, the median (range) age of patients was 48 (4–86) years. Of 90 patients where information was provided, 57% were female. In at least 28% of patients, a genetic cause was identified. Common presenting features include abdominal pain, palpable mass, cardiovascular and gastrointestinal symptoms. The most common tumour component with phaeochromocytoma is ganglioneuroma; other components include ganglioneuroblastoma, neuroblastoma and malignant peripheral nerve sheath tumours. In patients with follow-up data (*n*=48), 85% of patients were alive and well at a median (range) follow-up time of 18 (0.5–168) months.

**Conclusion:**

Composite phaeochromocytoma is a rare tumour, with a significant genetic predisposition. This review summarises available epidemiological data, which will be useful for clinicians managing this rare condition.

## Introduction

Phaeochromocytomas are chromaffin cell tumours characterised by the excessive production and secretion of catecholamines. These tumours usually arise in the adrenal medulla but occasionally from chromaffin cells of the sympathetic ganglia; here they are called paragangliomas. These tumours occur in approximately 0.5–2 patients per 1000 with hypertension [1]. Diagnosis is typically made between the third and fifth decades; up to 30% of phaeochromocytomas have a genetic predisposition including syndromes such as von Hippel-Lindau syndrome, neurofibromatosis type 1 (NF-1) and multiple endocrine neoplasia (MEN) syndrome type II [2, 3].

Approximately 15% of phaeochromocytomas are malignant [4]. Patients may present with sustained or paroxysmal hypertension, with associated symptoms such as headaches, sweating, palpitations and tremor [4], caused by the excessive release of catecholamines. Initial biochemical investigations include plasma and/or urine catecholamine and metanephrine levels. If these are elevated, imaging to locate a potential tumour includes CT and/or MRI scans. If these scans are inconclusive, nuclear medicine imaging using radiotracers ^123^I-metaiodobenzylguanidine (MIBG scan) [1] and more recently gallium-68 dotatate PET (^68^GA-PET) scans may also be useful [5, 6].

Surgery (usually by laparoscopy) after preoperative alpha blockade is the recommended intervention. Alpha blocker therapy is traditionally used reduce the risk of perioperative cardiovascular complications [1, 7]. The prognosis after surgery is very good for benign tumours; the 5-year survival rate is 95%; however, this drops to 50% in malignant tumours [1]. Treatment of metastatic disease is not well understood due to tumour rarity. Chemotherapy, radionuclide agents such as iobenguane ^131^I, tyrosine kinase inhibitors such as sunitinib and immunotherapeutic agents such as pembrolizumab have been reported; however, clinical trials for these treatments are ongoing [8]. Long-term follow-up is important in both benign and malignant pathology to ensure that recurrences are detected promptly [1].

Ganglioneuromas are rare tumours of autonomic ganglion cells of the nervous system. They are usually benign and often arise in the posterior mediastinum and retroperitoneum. They rarely occur in the adrenal gland, accounting for 0.3–2% of all adrenal incidentalomas [9]. Most adrenal ganglioneuromas are discovered incidentally on CT scans as they are largely asymptomatic and hormonally inactive. However, 30% of patients with ganglioneuromas are found to have raised plasma and urinary metanephrines [10]. Diagnosis can only be confirmed on histology after resection and prognosis is extremely good [9, 10].

Rarely, phaeochromocytomas may be part of a composite tumour [11], where there is another type of tumour (usually of the same embryological origin, i.e. neural crest) present. Tumour types that co-exist with phaeochromocytomas are reported to include ganglioneuromas, schwannomas and ganglioneuroblastomas [12, 13]. There are only a few documented cases in the literature, leading to uncertainty in the understanding of the pathogenesis and natural history of these conditions.

The aim of this study was to report cases of composite phaeochromocytomas seen in one centre and to undertake a systematic review of the published literature to increase the understanding of the epidemiology and clinical outcomes of these rare tumours.

## Methods

The histology reports of all patients who underwent resection of phaeochromocytoma over a 19-year period were reviewed to identify patients with composite tumours (defined as a tumour including phaeochromocytoma as at least one of several components). Two patients were identified in a review of 115 reports.

A systematic review of literature was performed to identify all reports of patients with composite tumours including phaeochromocytoma. The online database Medline was searched (on April 16, 2020), via search engine PubMed using the following combination of keywords:

- Phaeochromocytoma
- Composite OR combined OR incidental OR complex OR co-existing OR coincidental OR associated
- Paraganglioma OR ganglioneuroma OR neurofibroma OR schwannoma OR ganglioneuroblastoma OR neurilemmoma OR neuroendocrine carcinoma

The titles and/or abstracts of all articles retrieved by the search were reviewed independently by two authors to include original human studies on patients with composite tumours with phaeochromocytoma as one component. Studies on non-composite tumours, animal studies and those not written in the English language were excluded.

Full texts of articles considered suitable for inclusion were reviewed against the same criteria. The bibliography of included papers was also screened. Data from all included studies on demographics, clinic-pathological features and outcomes were collected in an excel spreadsheet and analysed. Included studies were also critically appraised using the Joanne Briggs Institute (JBI) critical appraisal checklist for case reports [14]. One point was awarded for each question if appropriate. Scores were reported as a percentage of the total applicable questions.

Descriptive analyses included reporting of frequencies (or percentages) for categorical data; median (range) for nonparametric, continuous data and mean (SD) for parametric, continuous data.

As this was a systematic review of literature and presentation of case studies where all identifiable details have been removed, no formal permission has been obtained from the research department and patient consent was not deemed necessary.

## Results

### Case presentations

#### Case 1

**Fig. 1:**
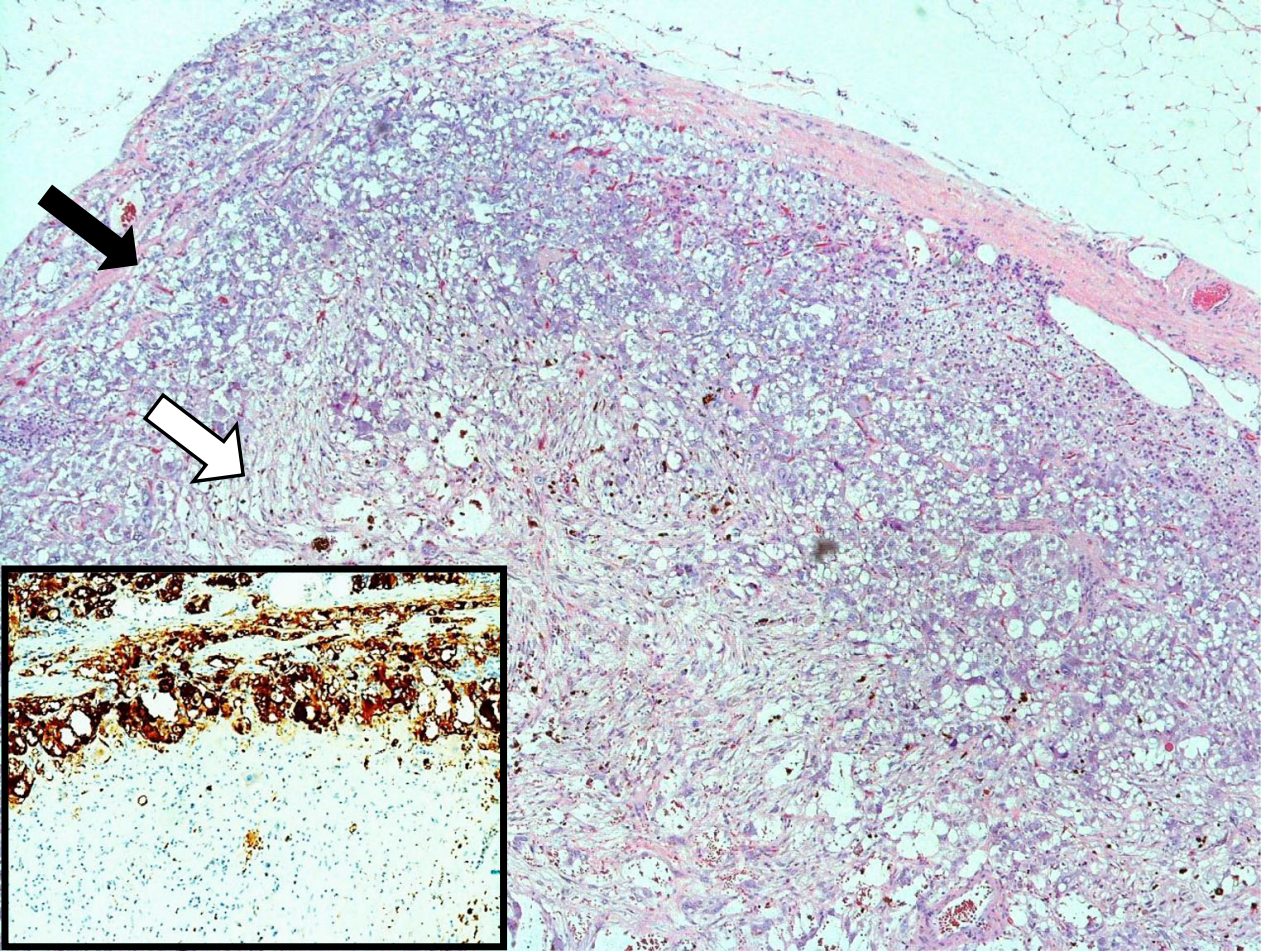
Low power view (×4) showing pheochromocytoma (solid black arrow) and ganglioneuroma (solid white arrow) as part of the composite phaeochromocytoma. The inset shows chromogranin A staining at ×10 magnification

A 69-year-old gentleman with learning difficulty, hypertension and neurofibromatosis type I presented in 2019 with haematuria. Abdominal examination only showed some neurofibromas and a café au lait lesion. Investigations revealed no definite cause, but CT scan of the urinary tract demonstrated left (4.5 cm) and right (2 cm) adrenal nodules. Biochemical testing demonstrated raised plasma and urine metanephrines, confirming a diagnosis of phaeochromocytoma. I-123 MIBG scans demonstrated uptake in both glands, but more on the left side. Following discussions in a multi-disciplinary team meeting, the patient and his family, left adrenalectomy alone was considered to avoid life-long steroid treatment. Regular surveillance of metanephrine levels was considered preferable to steroid treatment, as compliance with medications was an issue. After preoperative alpha blockade and with perioperative steroids, a left laparoscopic adrenalectomy was performed uneventfully. He was discharged on a low dose of phenoxybenzamine and bisoprolol. Histology showed features of a composite phaeochromocytoma-ganglioneuroma (Fig. 1) with a PASS score of 5/20. The patient and his metanephrine levels 19 months after surgery were satisfactory.

#### Case 2

**Fig. 2:**
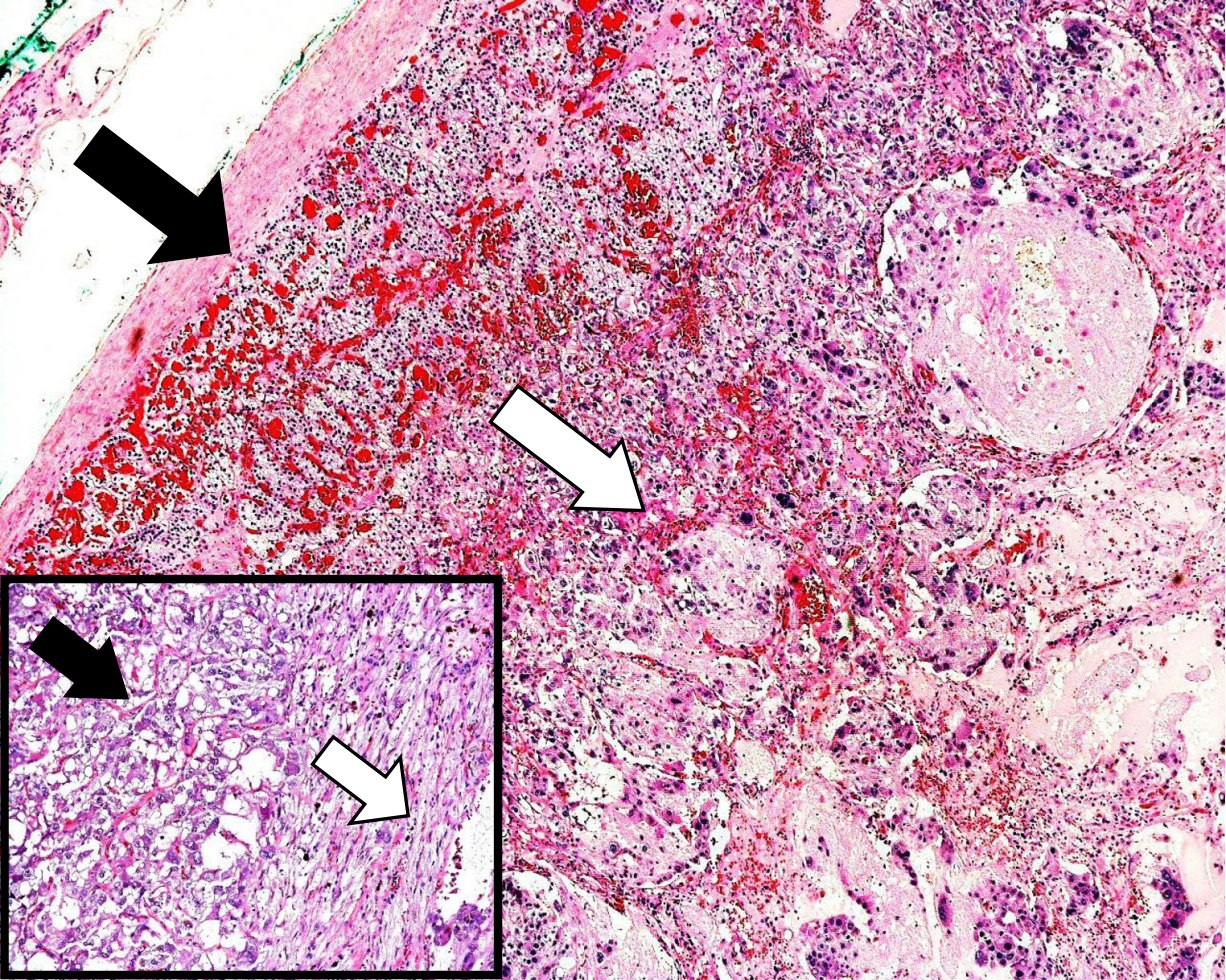
Low power view (×4) with background adrenal (solid black arrow) in the left upper part of the picture and composite pheochromocytoma on the right. The inset is a high-power view (×10) showing pheochromocytoma on the left (solid black arrow) and ganglioneuroma on the right (solid white arrow)

**Fig. 3:**
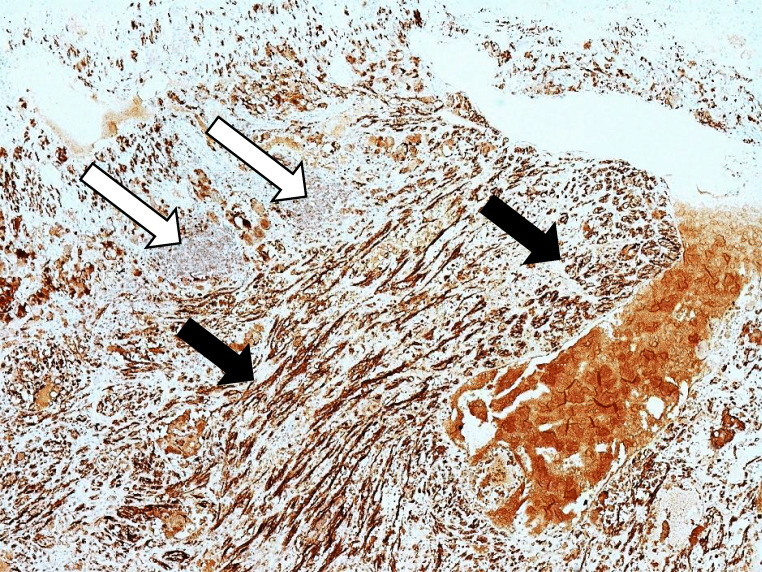
Low power view (4x) showing S100 staining highlighting the Schwann cells and the sustentacular cells of the composite pheochromocytoma (solid black arrows) but sparing the ganglion cells (solid white arrow) of the ganglioneuroma

A 75-year-old gentleman was incidentally found to have a 6-cm left adrenal lesion on CT scan for a new diagnosis of prostate cancer. He had hypertension and gout. A further fluorodeoxyglucose (FDG) PET scan demonstrated raised uptake and biochemistry confirmed raised metanephrine levels, confirming the diagnosis of phaeochromocytoma. After alpha blockade, the patient underwent an uneventful laparoscopic left adrenalectomy. Histology showed a composite tumour with two components—phaeochromocytoma and ganglioneuroma—with a PASS score of 4/20 (Figs. 2 and 3). He was well at 25 months following surgery.

Data from these two cases are summarised in Table 1.

**Table 1:** Summary of two cases from host institution

	Case 1	Case 2
Gender	Male	Male
Age at presentation (years)	69	75
Composite tumour component	Ganglioneuroma	Ganglioneuroma
Underlying genetic syndrome	NF-1	None
Presentation details	Haematuria	Incidental finding
Imaging modality	CT, MIBG	CT, FDG-PET
Primary management	Laparascopic adrenalectomy	Laparascopic adrenalectomy
Tumour diameter (cm)	4.5	6
Tumour laterality	Left	Left
Tumour PASS score	5/20	4/20
Outcome	Alive without disease	Alive without disease
Follow-up (months)	19	25

**Fig. 4:**
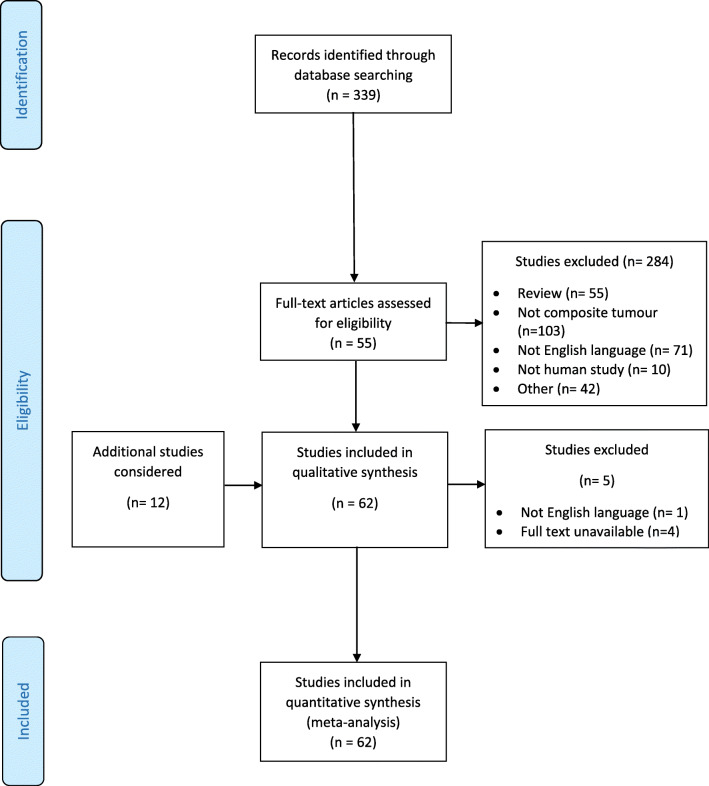
Modified PRISMA flow diagram showing the process of inclusion and exclusion of articles included in this review

**Fig. 5:**
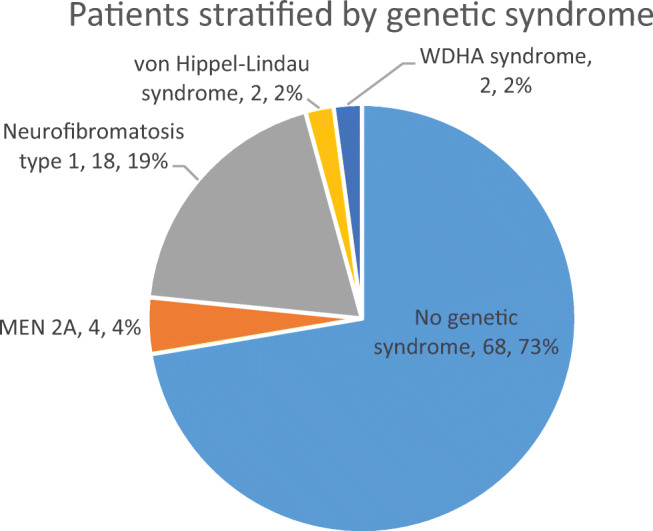
Pie chart showing patients with composite phaeochromocytoma in the review stratified by underlying genetic syndrome

**Fig. 6:**
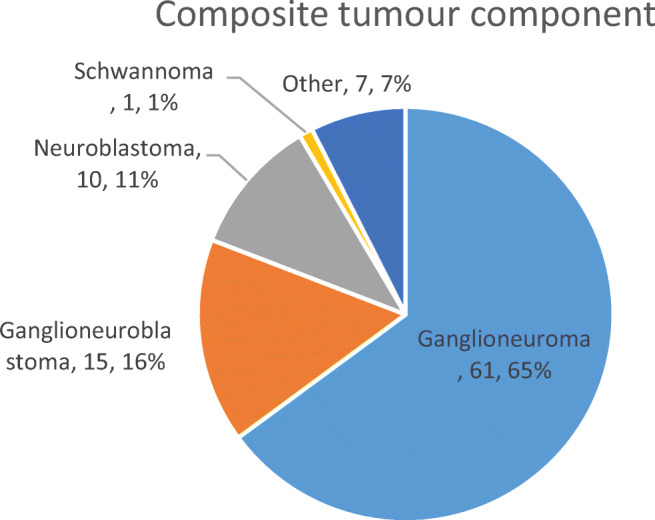
Pie chart showing patients with composite phaeochromocytoma included in the review, stratified by composite tumour components

The process of inclusion and exclusion of articles for this review is shown in a modified PRISMA flow diagram (Fig. 4). In total, 62 studies published between the years 1943 and 2017 involving 94 patients were included [15–73]. Of these studies, 51 were single-case reports. Of the 90 patients where gender information was available; there were 39 males (42%) and 51 females (54%). The median (range) age of patients where this information was available (*n*=91) was 48 (4–86) years. Data on patient demographics, tumour size and histology, underlying genetic syndrome and presentation details is presented in Table 2. Data on genetic syndromes and on composite tumour components are displayed in Fig. 5 and Fig. 6, respectively. Data on imaging modalities used, primary management, tumour laterality and removal method, patient outcomes and follow-up times is displayed in Table 3.

**Table 2:** Summary of demographic, clinical presentation, histology and underlying genetic syndrome in patients with composite phaeochromocytoma

Category (*n*=number of patients where data is available)	Classification	Number of patients
Gender (*n*=90)	Male	39 (42%)
	Female	51 (54%)
Age (*n*=91)	Median (range): 48 (4–86)	
Composite tumour component histology (*n*=94)	Ganglioneuroma	61 (65%)
	Ganglioneuroblastoma	15 (16%)
	Neuroblastoma	10 (11%)
	Schwannoma	1 (1%)
	Other*	7 (7%)
Underlying genetic syndrome (*n*=94)	None	68 (73%)
	NF-1	18 (19%)
	MEN 2A	4 (4%)
	von Hippel-Lindau syndrome	2 (2%)
	WDHA syndrome2	2 (2%)
Presentation details (*n*=74)	Incidental finding	35 (47%)
	Abdominal pain/palpable mass	24 (32%)
	Hypertension	18 (24%)
	Headaches	14 (19%)
	Weight loss	14 (19%)
	Diarrhoea	12 (16%)
	Palpitations and/or tachycardia	10 (14%)
	Nausea and/or vomiting	5 (7%)
	Sweating	4 (5%)
	Anxiety	3 (4%)
	Dysuria/haematuria	3 (4%)

*Other includes: “neuroendocrine carcinoma” (*n*=1), “ganglion cells in clusters” (*n*=1), “malignant peripheral nerve sheath tumour” (MPNST—*n*=3), “MPNST sustentaculoma” (*n*=1), “MPNST-rhabdomyosarcoma—Triton tumour” (*n*=1)

**Table 3:** Imaging modalities used, primary management and clinical outcomes of patients with composite phaeochromocytoma

Category (*n*=number of patients where data is available)	Classification		Number of patients
Imaging modality (*n*=57)	CT		45 (78%)—solitary modality in 24 (42%)
	MRI		15 (26%)
	MIBG		21 (37%)—with CT in 11 (19%), with MRI in 5 (9%)
	Ultrasound		4 (7%)
Primary management (*n*=74)	Adrenalectomy		66 (90)
	None—autopsy after death		6 (8%)
	Radiotherapy		1 (1%)
	Chemotherapy (for neuroblastoma metastases) + adrenalectomy		1 (1%)
Tumour diameter (*n*=81)	Median (range): 4.98cm (1.5–26)		
Tumour laterality (*n*=59)	Left (*n*=23, 39%)	Laparoscopic	4 (7%)
		Unspecified	19 (32%)
	Right (*n*=33, 56%)	Laparoscopic	5 (8%)
		Unspecified	28 (48%)
	Bilateral (*n*=3, 5%)	Laparoscopic	3 (5%)
		Unspecified	8 (14%)
Outcomes (*n*=70)	Alive without disease		55 (79%)
	Alive with metastases		1 (1%)
	Death from composite tumour		10 (14%)
	Death from other disease*		4 (6%)
Follow up time (*n*=48)	Median (range): 18 months (2 weeks–14 years)		
Time between primary management and death (*n*=7)	Median (range): 8 months (3–168 months)		

*Other causes of death include colorectal cancer (*n*=1), non-small cell lung cancer (*n*=1), bladder cancer (*n*=1) and myocardial infarction (*n*=2)

Left-sided operations were performed in 23 instances with 4 specifically described as laparoscopic procedures; the remaining 19 were unspecified. Right-sided operations were performed in 33 patients, with 5 specifically described as laparoscopic procedures; the remaining 28 were unspecified. In 8 procedures, the laterality was not mentioned. Three of these procedures were explicitly described as laparoscopic; the remaining 5 were unspecified. In 3 patients, bilateral resections were performed; one was explicitly described as an open method, while the remaining two were unspecified.

In 48 patients where follow-up information was available, most (85%) patients remained alive without disease at follow-up. Follow-up times ranged from 2 weeks to 14 years, with a median value of 18 months. The median (range) time of death for the 7 of 9 patients where information was available was 8 with a range of (3–168) months.

Of 62 studies, the median (range) score was 100% (range 0–100%); 41 scored 100% in the JBI classification (Table 4). There was a study that scored zero, which was a larger series of adrenal lesions that included 3 patients with composite tumours, but provided no relevant information [16].

**Table 4:** JBI critical appraisal of case reports

JBI Question No.	Score
1. Were the patients’ demographic characteristics clearly described?	61/62 (98%)*
2. Was the patient’s history clearly described and presented as a timeline?	53/61 (85%)
3. Was the current clinical condition of the patient on presentation clearly described?	55/61 (91%)
4. Were diagnostic tests or assessment methods and the results clearly described?	47/61 (77%)
5. Was the intervention(s) or treatment procedure(s) clearly described?	52/59 (88%)
6. Was the post-intervention clinical condition clearly described?	50/58 (86%)
7. Were adverse events (harms) or unanticipated events identified and described?	27/30 (90%)
8. Does the case report provide takeaway lessons?	58/62 (94%)

E.g. of the 62 studies for which question 1 was applicable, 61 studies satisfied the question (98%)

## Discussion

Composite phaeochromocytoma tumours are extremely rare [9]. Two patients were identified in this unit over a 19-year period, in addition to the 94 patients identified in this review.

Genetic syndromes including neurofibromatosis 1, MEN 2A, von Hippel Lindau syndrome and watery-diarrhoea hypokalaemia-achlorhydria (WDHA) syndrome were only identified in 28% of patients, similar to patients with phaeochromocytoma alone—a review of 314 patients with phaeochromocytoma 27.4% with an underlying genetic cause [74].

The pathogenesis of composite tumours is unclear. Apart from coincidental occurrence, alteration in the microenvironment of one tumour may favour the formation of a second tumour arising in the same area [75].

Although not currently included in treatment guidelines [7], recent small studies have shown ^68^Ga-dotatate PET scans to be more specific than MIBG in the diagnosis of phaeochromocytoma and paraganglioma (PPGL) tumours [5, 6]. However, this imaging modality is not widely available.

Of 94 case reports, only seven reported use of alpha blocker therapy before surgery. Alpha blocker therapy pre-surgery is currently recommended in all patients with functional phaeochromocytoma-paragangliomas (PGGLs) to reduce the risk of hypertensive crisis [7]. However recent studies have shown that alpha blockade may not have any effect on intraoperative blood pressure or mortality [76–79].

This review is fairly comprehensive, but it is not possible to make clear recommendations on management based on a review of case studies. Another limitation of our review is the discrepancy in reporting quality amongst the case reports.

## Conclusion

This review provides comprehensive demographic and clinical information on composite phaeochromocytomas published in the literature. These tumours affect men and women equally, with the majority of diagnoses occurring between the third and fifth decades. Clinical presentation can be classified into two main categories: cardiovascular and gastrointestinal. Adrenalectomy is the gold standard treatment and prognosis is good; however, these tumours remain extremely rare and their occurrence should prompt consideration for genetic testing.
